# Any Place for Immunohistochemistry within the Predictive Biomarkers of Treatment in Lung Cancer Patients?

**DOI:** 10.3390/cancers10030070

**Published:** 2018-03-13

**Authors:** Véronique Hofman, Sandra Lassalle, Coraline Bence, Elodie Long-Mira, Sacha Nahon-Estève, Simon Heeke, Virginie Lespinet-Fabre, Catherine Butori, Marius Ilié, Paul Hofman

**Affiliations:** 1 Laboratory of Clinical and Experimental Pathology, Côte d’Azur University, FHU OncoAge, Pasteur Hospital, 30 Avenue de la voie Romaine, 06001 Nice CEDEX 01, France; hofman.v@chu-nice.fr (V.H.); lassalle.s@chu-nice.fr (S.L.); bence.c@chu-nice.fr (C.B.); long-mira.e@chu-nice.fr (E.L.-M.); nahon-esteve.s@chu-nice.fr (S.N.-E.); heeke.s@chu-nice.fr (S.H.); lespinet-fabre.v@chu-nice.fr (V.L.-F.); Butori.c@chu-nice.fr (C.B.); ilie.m@chu-nice.fr (M.I.); 2 Hospital-Integrated Biobank (BB-0033-00025), Pasteur Hospital, 30 Avenue de la voie Romaine, 06001 Nice CEDEX 01, France

**Keywords:** predictive biomarkers, lung cancer, immunohistochemistry, immunocytochemistry, immune-oncology

## Abstract

The identification of certain genomic alterations (*EGFR*, *ALK*, *ROS1*, *BRAF*) or immunological markers (PD-L1) in tissues or cells has led to targeted treatment for patients presenting with late stage or metastatic lung cancer. These biomarkers can be detected by immunohistochemistry (IHC) and/or by molecular biology (MB) techniques. These approaches are often complementary but depending on, the quantity and quality of the biological material, the urgency to get the results, the access to technological platforms, the financial resources and the expertise of the team, the choice of the approach can be questioned. The possibility of detecting simultaneously several molecular targets, and of analyzing the degree of tumor mutation burden and of the micro-satellite instability, as well as the recent requirement to quantify the expression of PD-L1 in tumor cells, has led to case by case development of algorithms and international recommendations, which depend on the quality and quantity of biological samples. This review will highlight the different predictive biomarkers detected by IHC for treatment of lung cancer as well as the present advantages and limitations of this approach. A number of perspectives will be considered.

## 1. Introduction

Technological progress, the discoveries made by fundamental research laboratories and the development of translational research performed in particular thanks to biobanks, as well as the availability of precisely annotated clinical samples have resulted in the emergence of biomarkers associated to novel therapeutics for patients with lung cancer [1,2,3,4]. While it is now mandatory to look for some of these biomarkers, since they are associated to the efficacy of validated treatments and are accessible, other biomarkers are evaluated in therapeutic trials, as well as in clinical and exploratory research protocols [5,6,7,8]. When considering biomarkers of interest, the sensitivity and specificity of the tests aimed at their identification must be evaluated and compared. Participation in external evaluation of the quality is a prerequisite before implementation by a laboratory of predictive biological tests for therapeutic response [9,10,11]. When considering the therapeutic issues, the benefit-risk balance for patients and the management of economic models related to public health, it is essential to use a reliable biomarker [12]. 

The increase in the number of biomarkers for evaluation in a patient with late stage or metastatic lung cancer raises several problems: (*i*) Is it possible to look sequentially for several biomarkers? (*ii*) Should the panels be used systematically at a single time? (*iii*) Can immunohistochemistry (IHC) or molecular biology (MB) methods be combined using small-sized tissue samples or cytological samples?

This review will summarize the main therapeutic targets detected with IHC for care of patients with non-small cell lung cancer (NSCLC) or small cell lung cancer (SCLC). It will specify the circumstance under which this approach is appropriated for personalized medicine and compare it to the possibilities proposed by techniques of MB.

## 2. Tissue and Cell Samples and the Management of Daily Practice

**Figure 1:**
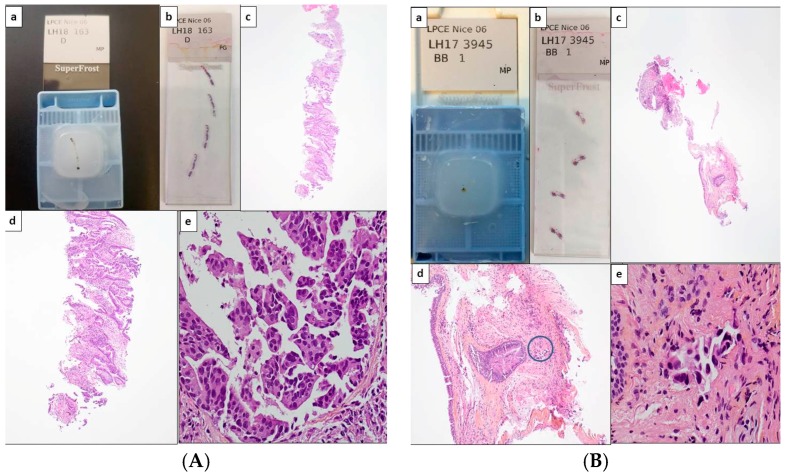

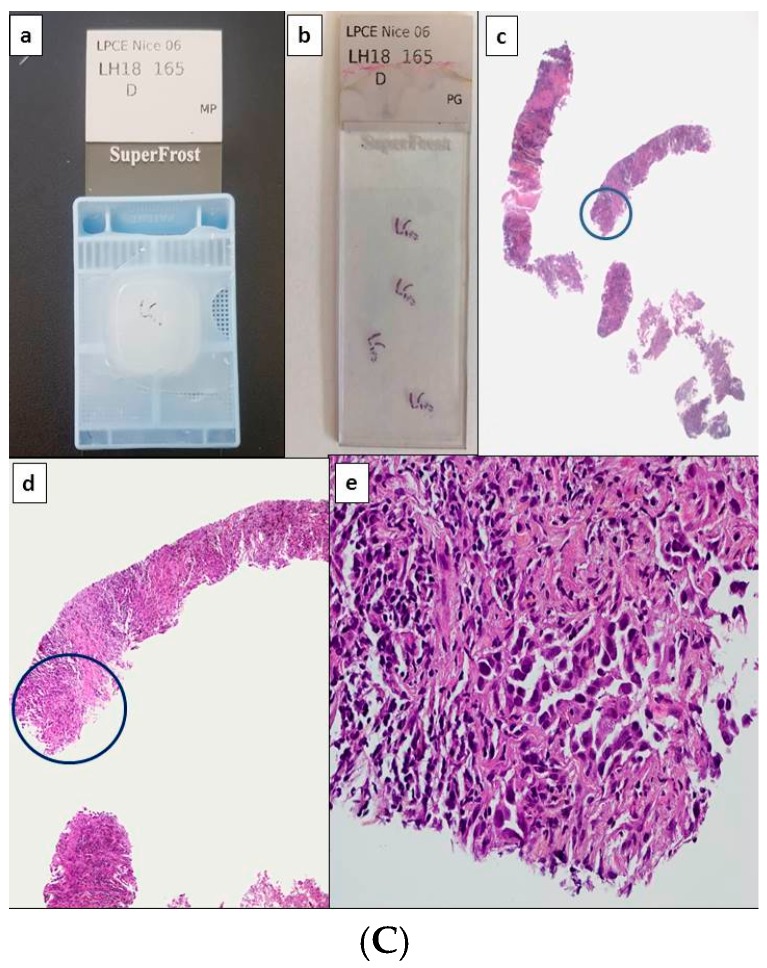
Potential scenarios for detection of theranostic biomarkers for lung adenocarcinomas using thoracic biopsies. Depending on the quantity of tumor cells detected on hematoxylin and eosin stained sections, immunohistochemistry (IHC) and/or molecular biology approaches can be used (with or without panels). (**A**) Large biopsy with a high percentage of tumor cells (more than 50%). Paraffin block with one biopsy (**a**) and corresponding 4 tissue sections stained with hematoxylin eosin (**b**). Different magnifications of the same tissue section showing high number of adenocarcinoma cells (**c**–**e**). In this latter case, the number of tumor cells should allow PD-L1 IHC to be performed first and then followed by NGS. Alternatively a workflow including successive PD-L1, ALK, ROS1, and BRAF IHC, and then NGS can be also adopted. (**B**) Small biopsies with a few tumor cells. Paraffin block with small biopsies (**a**) and corresponding 4 tissue sections stained with hematoxylin eosin (**b**). Different magnifications of the same tissue section showing in only one area (circle) a low number of tumor cells (no more than 15 tumor cells) (**c**–**e**). IHC targeting PD-L1, then IHC for EGFR, ALK, ROS1, and BRAF should be probably done. (**C**) Large biopsies with a low percentage of tumor cells (less than 5%). Paraffin block with at least 5 large biopsies (**a**) and corresponding 4 tissue sections stained with hematoxylin eosin (**b**). Different magnifications of the same tissue section showing in only one area (circle) some tumor cells (**c**–**e**). IHC for PD-L1 then successively for ALK, ROS1 and BRAF can be done and followed by targeted molecular biology for detection of EGFR mutations on a single tissue section or alternatively IHC for EGFR, according to the sensitivity of the MB test [1Ac, 1Bc, 1Cc, original magnification ×25; 1Ad, 1Bd, 1Cd, original magnification ×100; 1Ae, 1Be, 1Ce, original magnification ×400].

The techniques for getting tissue and cytological specimens that provide diagnosis of lung cancers have evolved, have become less invasive and now also evaluate the metastatic stage, in particular for mediastinal lymph nodes [13,14,15,16]. These techniques give more and more distal access to small lesions of the bronchial tree [13,14,15,16]. However, it became progressively apparent that one of the constraints of these new methods was that they provided small and fragmented amounts of tissue instead of larger and complete tissue samples. Thus, when conducting morphological and/or molecular approaches the available morphological material must be considered [17,18] (Figure 1). The sensitivity of the detection method of the different molecular targets, as well as their specificity, mean that, depending on the case, one of these methods cannot be performed or else reservation concerning the solidity or reliability of the results may be issued. Beyond the nature and quantity of the available biological material, pre-analytical steps must control the type and the fixation time due to their impact on the quality of the nucleic acids and proteins [19,20]. Aside from the length of the cold ischemia (delay between sampling and fixation), which is generally very short for thoracic biopsies, the pre-analytical steps must master the type and the time duration of fixation while taking into consideration their impact on the quality of the nucleic acids and proteins [19,21]. Thus, the use of solutions other than formol is not recommended for IHC since the degradation of certain epitopes makes the interpretation of the detection of different signals inexact [19,21]. 

Of particular importance, alcohol fixation of cytological samples for ICC gives variable results depending on the antibody and the epitope for identification [22]. This type of fixation is not recommended in the absence of comparative analyses and validation with samples treated with formol. The time of fixation with formol must be controlled. In particular, hypofixation of a tissue biopsy (in general less than 6–8 h) can have a substantial impact on the results of IHC. Inappropriate fixation has also important consequences on the quality of the molecular analyses [fluorescence in situ hybridization (FISH) or sequencing techniques]. The degradation of nucleic acids (DNA and RNA) after over- or under-fixation or fixation with a solution other than formol makes the MB techniques noncontributory [23]. Finally decalcification/pre-treatment conditions used before bone tissue sectioning are usually unsatisfactory for IHC as well as for MB approaches [19].

## 3. The Therapeutic Targets Identified by Immunohistochemistry in Thoracic Oncology

**Figure 2:**
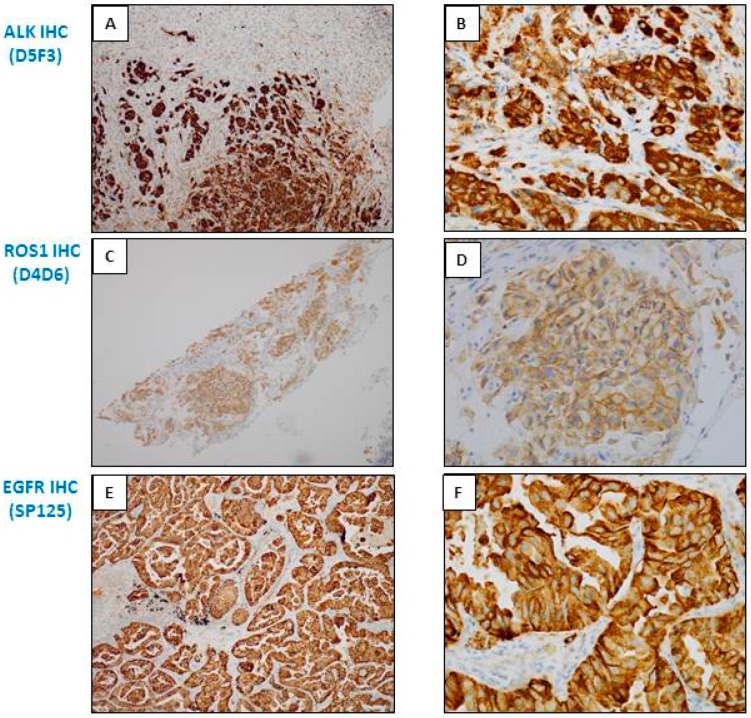

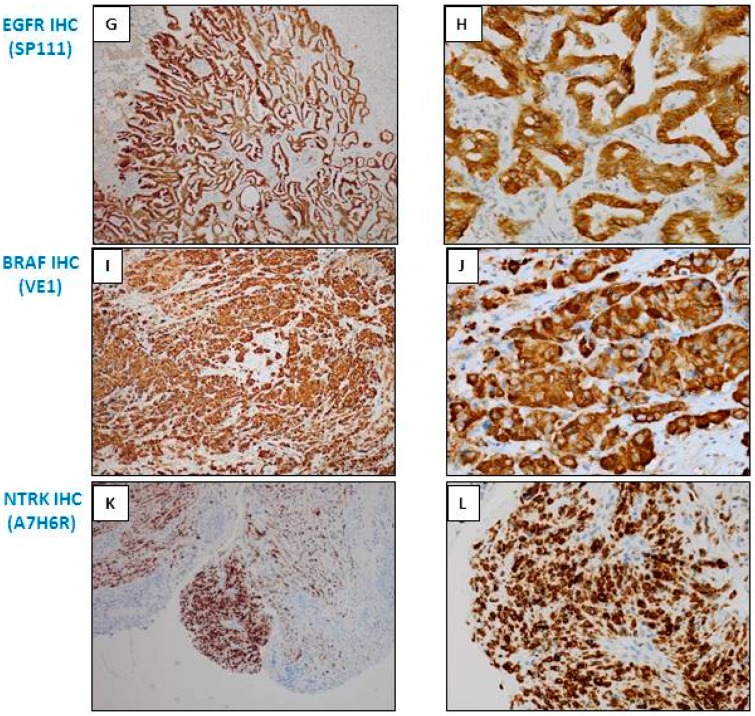
Examples of different types of staining of immunohistochemistry (IHC) obtained with bronchial biopsies with antibodies used as theranostic tools for lung adenocarcinoma. (**A**,**B**) ALK IHC (D5F3, Ventana); (**C**,**D**) ROS1 IHC (D4D6, Cell Signaling); (**E**,**F**) EGFR IHC (L858 EGFR mutation; SP125, Ventana); (**G**,**H**) EGFR IHC (del 19 EGFR mutation; SP111, Ventana); (**I**,**J**) BRAF V600E IHC (VE1, Ventana); (**K**,**L**) pan Trk IHC (A7H6R, Cell Signaling). (**A**,**C**,**E**,**G**,**I**,**K**) Immunoperoxidase, magnification ×100; (**B**,**D**,**F**,**H**,**J**,**L**) Immunoperoxidase, magnification ×400.

**Figure 3:**
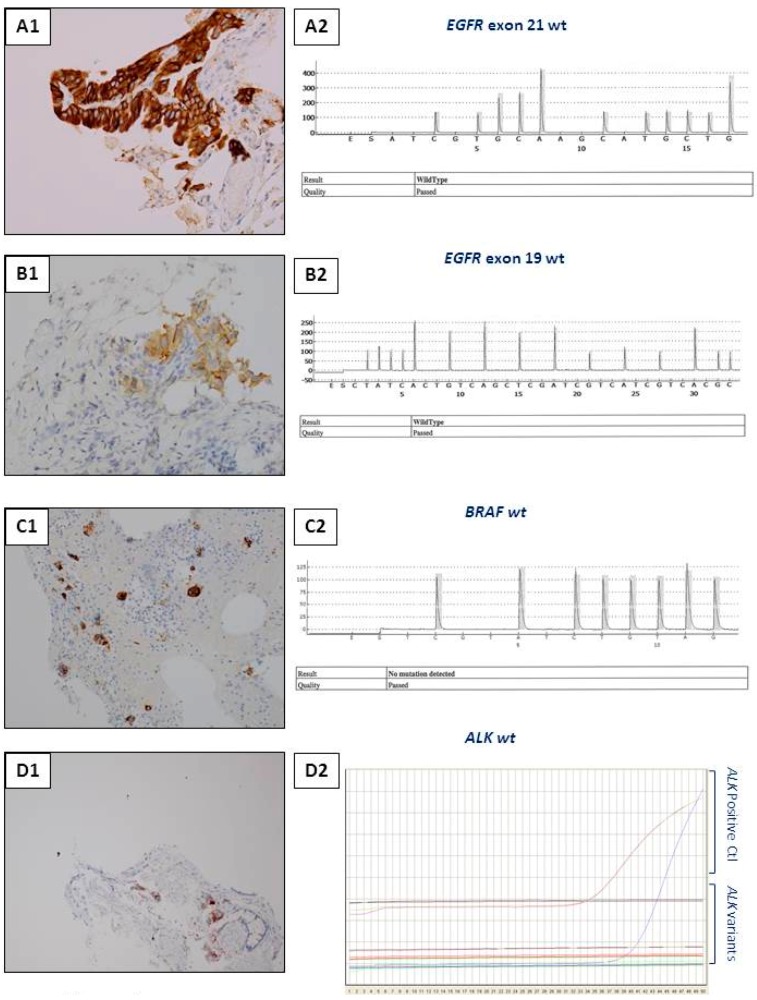
Examples of interest for use of IHC as a unique tool for theranostic biomarker detection in biopsies with a few tumor cells. (**A**) L858R *EGFR* mutation detection. (**A1**) Positive EGFR IHC using the SP125 clone. (**A2**) A negative molecular biology result (using a pyrosequencing/therascreen *EGFR* panel from Qiagen, Hilden, Germany) for detection of the L858R *EGFR* mutation with the same biopsy. (**B**) Detection of the Del 19 *EGFR* mutation. (**B1**) Positive EGFR IHC using the SP111 clone. (**B2**) A negative molecular biology result (using a pyrosequencing/therascreen *EGFR* panel from Qiagen, Hilden, Germany) for the Del 19 mutation in *EGFR* detected in the same biopsy. (**C**) Detection of the *BRAF V600E* mutation. (**C1**) Positive BRAFV600E IHC using the VE1 clone. (**C2**) A negative molecular biology result (using a pyrosequencing/therascreen *BRAF* panel from Qiagen) for detection of a *BRAF* mutation. (**D**) *ALK* status detection. (**D1**) *ALK* status assessment. (**D1**) A positive ALK IHC using the D5F3 clone. (**D2**) A negative molecular result for detection of a *ALK* rearrangement [using RT-PCR targeting the variants 1 (E13; A20), 2 (E20; A20), 3A and 3B (E06; A20), 5 (E02; A20) and 7 (E17; A20) of *ALK*].

Several therapeutic targets can be detected in the daily practice using antibodies for IHC. The anti-ALK, anti-ROS1, anti-EGFR mutated, anti-BRAF V600E, anti-NTRK, and anti-PD-L1 can potentially be used by pathologist on tissue material (bronchial or transthoracic biopsies in particular) (Figure 2 and Figure 3). The use of these antibodies with formol fixed cytological material requires validation steps and comparative approaches with results obtained with tissue biopsies performed preferably on the same patient. In general, the cytological samples examined after formol fixation and inclusion into paraffin blocks (cytoblocks) give similar results to those obtained with tissue samples [24,25,26,27]. The other cytological approaches (absence of fixation, fixation other than with formol, cytological smears, cytology after centrifugation onto slides) make standardization of ICC approaches difficult. The sensitivity of the different antibodies is generally very good in the event of appropriate management of the pre-analytical phases. Their specificity must be considered and controlled according to the clone for use. Some clones are used only for companion diagnostic tests approved by the Food and Drug Administration (FDA) in the USA and have thus been validated in comparison with the efficacy of the administrated therapeutic molecule, while other clones are evaluated in laboratories as laboratory developed tests (LDT) and require strong caution for clinical validation [28].

### 3.1. Anti-ALK Antibodies

These antibodies are routinely used in most pathology laboratories [29]. The D5F3 clone (Ventana, Tucson, AZ, USA) is a companion diagnostic test validated by the FDA [30,31]. It is often used and its sensitivity and specificity are excellent [32]. However, it should only be used with the Ventana platform (Roche Diagnostic, Tucson, AZ, USA) and its cost can be higher than other clones. Modification can be required when using the Benchmark Ultra (Ventana) compared to the Benchmark XT (Ventana), where some non-specific labeling can be visible (personal data). However, the D5F3 clone can be used in other IHC platforms than the Ventana platforms and as a laboratory developed test (LDT). The other clone the most often used is the 5A4 clone (Abcam, Cambridge, UK) [33]. Discordant results are sometimes obtained, depending on the study, some of which reveal lower sensitivity and less specificity than the D5F3 clone [30]. Other anti-ALK antibodies have been developed, but the tests of comparative and multi-center validation are strongly recommended before use in the routine clinic [34]. Thus, disregarding the clone for use quality controls must be performed before performing ALK IHC in the daily practice, knowing the therapeutic consequences associated to the obtained results [35,36,37]. Moreover, since these ALK IHC are rarely positive in NSCLC it must be mandatory to use ALK positive and negative samples as controls in parallel. While the FISH technique represented for a longtime the gold standard approach for the analysis of the *ALK* status, and a positive result on IHC required systematic validation of the status by FISH, recent recommendations indicate than intense labeling with the clone D5F3 on IHC is now sufficient for treatment of the patient with ALK inhibitors [34]. This is all the more important given that FISH *ALK* is more sensitive that ALK IHC to pre-analytical variables (in particular to poor fixation) and can turn out to be negative due to nucleic acid modification [38]. However when a weak or a moderate labeling with the clone D5F3 is observed, *ALK* FISH must be done in order to confirm the results. The use of anti-ALK antibodies for ICC is possible if the above precautions are observed [39,40]. At present, confirmation by FISH is required if a positive ALK result is obtained by ICC. One of the limitations of FISH for *ALK* on cytological samples is the number of tumor cells, which is sometimes less than 100 thereby making difficult the analysis as a function of the required cut off [40].

### 3.2. Anti-ROS1 Antibodies

Only a few anti-ROS1 antibodies have been validated and recommended for use in daily routine practice [41,42,43,44]. The D4D6 (Cell Signaling Technology, Leiden, The Netherlands) clone is used by the majority of laboratories [38]. The signal must be interpreted with care because certain cases are marginally positive and in general the label is not as intense as the one observed for anti-ALK antibodies. It is now essential in all cases to confirm rearrangement of *ROS1* by a FISH approach [45]. 

### 3.3. Anti-EGFR Mutated Antibodies 

Different antibodies targeting a mutation in *EGFR* can be used on fixed tissues [33,46,47,48,49,50,51,52]. Nonetheless the sensitivity of these antibodies is globally lower than MB methods, in particular those for detection of deletion in exon 19, and their specificity depends on the clone and the mutation for consideration [47,53]. One of the advantages of IHC is the identification of a molecular target on only a few cells (as for example those visible on development of a carcinomatous lymphangitis diagnosed with a biopsy). In this situation, the extraction of DNA does not lead to a sufficient quantity of somatic DNA for detection by MB methods for mutations in *EGFR* (Figure 3). Aside from the limits in sensitivity and specificity the main pitfall of IHC is the limited number of activating mutations of *EGFR* that can be detected, which consequently may not allow treatment of patients presenting with certain mutations that are sensitive to tyrosine kinase inhibitors. Another pitfall concerns the need to perform IHC analyses on several consecutive tissue sections for detection of the L858R mutation and deletion 19 in exon 21. Finally, no commercial antibody can detect resistance mutations in *EGFR* including the T790M and the C797S mutations. The advantages of IHC in comparison with MB approaches include primarily the rapidity with which the results are obtained, the lower cost and the widespread development of IHC in pathology laboratories. However some new MB techniques identify the mutational status of *EGFR* in a few hours in one tissue section and detect a greater number of mutations, which now strongly compete with the IHC approach [54]. 

### 3.4. Anti-BRAF Antibodies

Depending on the published series, 1 to 6 % of lung adenocarcinomas hold a mutation in the *BRAF* gene [55,56,57,58]. Only patients with a *BRAF V600E* mutation are eligible for targeted treatment. Several *BRAF* clones for IHC have been commercialized but the VE1 clone is particularly sensitive and specific for the detection of the *BRAF V600E* mutation [59,60]. This clone is of interest for use now that the commercialization of therapeutic molecules for administration to patients with metastatic lung adenocarcinomas carrying the *BRAF V600E* mutation has been approved. The status of this mutation can thus be detected with IHC on about ten tumor cells while the MB approach can be less efficient or negative after sequencing of DNA extracted from only a few tumor cells (Figure 3). The VE1 clone can recognize epitopes present on normal human cells, in particular ciliated cells, which requires rigorous interpretation of anti-BRAF IHC on certain bronchial biopsies [61].

### 3.5. Anti-PD-L1 Antibodies

**Figure 4:**
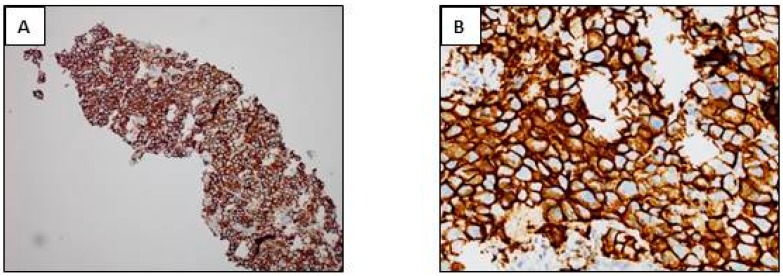

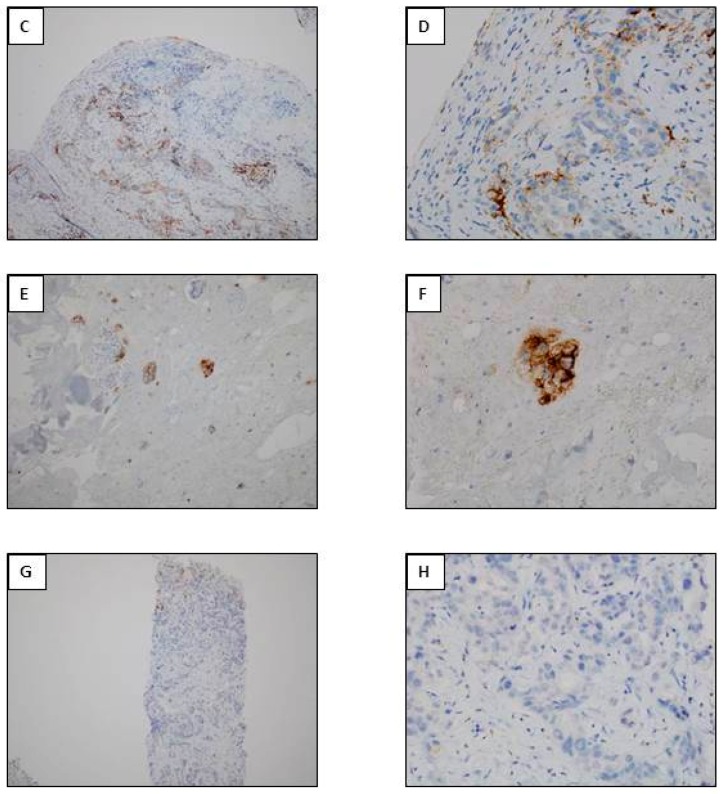
PD-L1 IHC (PD-L1 IHC 22C3 pharmDx, Agilent Technologies, Inc., Santa Clara, CA, USA). (**A**,**B**) Positivity of more than 50% of tumor cells. (**C**,**D**). Positivity of more than 1% and less than 50% of tumor cells. (**E**,**F**) Positivity of a few cells of a lymphangitis carcinoma. (**G**,**H**) Negative IHC. (**A**,**C**,**E**,**G**) immunoperoxisase, magnification ×100; (**B**,**D**,**F**,**H**) immunoperoxidase, magnification ×400).

The development of immune check point anti-PD1/PD-L1 inhibitors has radically modified the therapeutic strategy for advanced stage or metastatic NSCLC [62,63]. Conversely to the other antibodies described in this paper which can be used as a screening method or as an alternative method to MB, immunotherapy is based only on the level of expression of PD-L1 in tumor cells (Figure 4) [62]. The administration of the pembrolizumab molecule as first-line therapy to patients without genomic alterations in the genes *EGFR*, *ALK*, *ROS1* and *BRAF*, and for whom 50 % of the tumor cells express PD-L1, has given back IHC a primordial role in the therapeutic care of patients with NSCLC [63]. The development of anti-PD-L1 IHC is now done rapidly in most pathology laboratories, but all the steps require strict control [24,64,65,66,67]. The use of anti-PD-L1 IHC as a predictive biomarker for response to pembrolizumab therapy raises several questions. Many oncologists and pathologists consider this biomarker to be insufficient. In fact, some patients with a negative PD-L1 IHC show good response to immunotherapy [24,68]. In contrast, patients expressing strongly PD-L1 can show no response to anti-PD1/PD-L1 immunotherapy [24,68]. The heterogeneity in the expression of PD-L1 on IHC may be one of the reasons for these inconsistencies [69]. Moreover, PD-L1 expression varies in relation to histological patterns, with high levels in those with pleomorphic features and low expression in invasive mucinous adenocarcinomas and the lepidic components of non-mucinous adenocarcinomas [70]. In this context, evaluation of PD-L1 expression in some lung adenocarcinoma with a lepidic component may therefore be less reliable when considering immumodulatory therapy for recurrent disease. The question of which clone to use is also debatable [65,71]. So, should the kit PharmDx or LTD antibodies be used [65,66,71,72,73,74,75]? In this context, there are several commercially available PD-L1 IHC tests. These tests are designed by the clone used to detect the PD-L1 protein in tumor and/or immune cells. The 22C3 test (PD-L1 IHC 22C3 pharmDx, Agilent Technologies, Inc., Santa Clara, CA, USA) is currently the only test used as a companion diagnostic test (CDX) for the administration of pembrolizumab as first line treatement in advanced or metastatic NSCLC [73]. 

These CDX are tests with (per the US Food and Drug Administration definition) provide enough information that is essential for the safe and effective use of a corresponding drug or biologic product. Other tests such as IHC 28-8, SP142 and SP263 for nivolumab, atezolizumab and durvalumab, respectively are regarded as complementary diagnostics, but are not considered by the FDA as being essential for safe and effective treatment selection [73,74].

### 3.6. Anti-NTRK Antibodies

The detection of genomic alterations in *NTRK* has led to new therapeutic options for patients with lung cancer, in particular those with epidermoid carcinoma [76]. Anti-NTRK antibodies have been very recently developed and the expression of the protein has been shown to correlate with rearrangements in the gene [77]. Nonetheless, this molecular anomaly is very rare in NSCLC (less than 1% of patients) and so the usefulness of an IHC approach to evaluate the status of *NTRK* in this pathology is questionable. MB techniques, in particular next generation sequencing (NGS), can be more appropriate for detection of this rearrangement by its systematic evaluation concomitant to detection of other rearrangements in the genes *ALK*, *ROS1*, and *RET* [78]. However, NTRK IHC can be envisaged if only a very small number of tumor cells are visible in a biopsy and/or if the quality of the RNA proves to be inadequate for MB techniques.

### 3.7. Anti-RET Antibodies

The *RET* rearrangement has been evaluated for therapeutic trials using RET inhibitors [79,80,81]. Correlation between mutations, amplification and over-expression of RET have given conflicting results depending on the study [81,82].

### 3.8. Anti-MET Antibodies

The expression of the protein MET has been evaluated for phase I, II and III clinical trials evaluating the administration MET inhibitors [83]. An IHC score taking into consideration the percentage of stained tumor cells and the intensity of the label was defined. While the phase II was positive, the phase III of this therapeutic trial did not confirm the results and the MET IHC did not predict the therapeutic response [83,84]. The majority of the published studies used the SP44 clone [83,84]. Depending on the study, different correlations between the protein expression, the amplification detected by FISH and the evaluation of *MET* mutations were obtained [85,86,87,88]. Due to these inconsistencies MET IHC is not often used currently as a predictive approach to the response to *MET* targeted therapy. 

### 3.9. Anti-ERCC1 Antibodies

Initial studies into ERCC1 in metastatic lung epidermoid carcinomas showed good correlation between over-expression with IHC of this molecule and resistance to platinum salts [89,90,91]. The clone 8F1 was used [92]. Surprisingly these results were not confirmed when using the same clone [89,92]. The reason given was that the initial batch of antibody was not the same as that used subsequently and that it was impossible to reproduce the same quality of antibody. At present, ERCC1 IHC is not sufficiently robust for use as a predictive biomarker test of response to platinum salts for patients presenting with advanced stage or metastatic NSCLC [93,94].

### 3.10. Anti-DLL3 Antibodies

Until now, exceptional therapeutic trials concerning targeted therapy for SCLC were reported, given the absence of an associated biomarker for effector molecules. Treatment targeting the DLL3 molecule were recently shown to be more efficient than conventional treatment in phase I clinical trials for patients with SCLC strongly expressing the DDL3 protein on IHC [95,96,97].

### 3.11. Other Antibodies

A number of antibodies have been used for IHC in therapeutic trials. Thus, anti-PTEN, anti-LKB1 and anti-NRAS antibodies have been tested [98,99,100,101]. However, none of these antibodies have been used to date in the daily practice.

## 4. Integration of Molecular and Immunohistochemical Approaches: What Is the Future?

**Figure 5:**
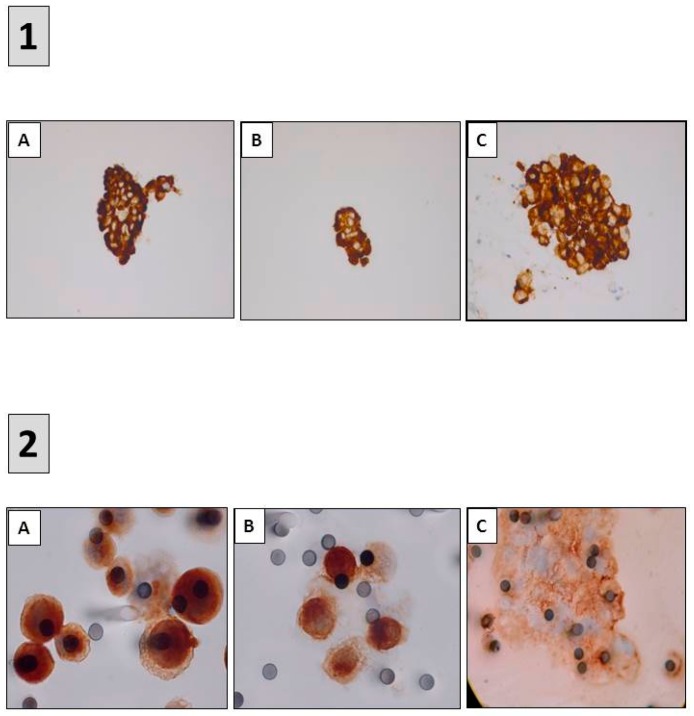
Detection of predictive biomarkers using immunocytochemistry (ICC). (**1**) ALK ICC (D5F3) (**A**) ROS1 ICC (D4D6) (**B**) and PD-L1 ICC (22C3) (**C**) performed on samples obtained after endobronchial ultrasound trans-bronchial needle aspirations (**A**–**C**) immunoperoxidase, magnification ×400. (**2**) ALK ICC (D5F3) (**A**) MET ICC (SP44) (**B**) and PD-L1 ICC (22C3) (**C**) performed on circulating tumor cells isolated on filters after blood filtration (**A**–**C**) immunoperoxidase, magnification ×1000.

MB techniques have improved considerably in recent years. These approaches are becoming more and more sensitive and require lower and lower amounts of nucleic acids. The methods of extraction of nucleic acids have also progressed and the yield of extraction from small-sized tissue or cytological samples has led to the development of targeted molecular analyses and, in particular, NGS using formaldehyde fixed biological material. Thus, the molecular targets for personalized therapy (in particular genomic alterations in the *ALK*, *ROS1*, *EGFR*, *BRAF*, *MET*, *HER2*, *NTRK*, and *RET* genes) can be investigated and detected, either in a sequential or targeted manner or simultaneously by analysis of different panels. In this context, the role of IHC in detecting certain anomalies is debatable and indications for analysis have been and will probably be fewer and fewer in pathology laboratories. However, a number of points can be discussed:

Despite the improvement in the technologies mentioned above, MB approaches can lead to negative or uncertain results for different reasons: (*i*) insufficient tissue, presence of necrotic areas and/or a low percentage of tumor cells, or a few cells will be only available for ICC (Figure 3 and Figure 5); and (*ii*) alterations in the nucleic acids due to poor fixation (over-/under-fixation) or the use of an inappropriate fixation solution other than buffered 10% formol [23]. Thus, depending on the team and the techniques (in particular NGS), the percentage of tumor cells, the size and quality of the samples the recorded level of failure with bronchial or transthoracic biopsies can be up to 25% for results from MB approaches. However, some publications demonstrated successful library DNA preparation and sequencing in a much higher percentage of cases [17,102,103]. In a more targeted manner, the evaluation of genetic rearrangement by NGS and by RT-PCR (in particular for the *ALK* or the *ROS1* rearrangement) requires a certain amount of good quality RNA, which can be absent for small-sized bronchial biopsies that contain a small number of tumor cells or are necrotic and/or are poorly fixed [104,105]. It is probable that initial ALK IHC is more useful in evaluating the *ALK* status than *ALK* MB approach. This is certainly true for detection of other molecular targets from a very few cells, as for the evaluation of the *BRAF V6000E* mutation with IHC compared to targeted molecular analysis or extended with a NGS type of approach [106]. 

In certain cases, when the results must be obtained in an urgent manner, some MB approaches, in particular NGS, and to a lesser extent targeted approaches, can sometimes delay administration of effective treatment. Only certain targeted MB approaches are now able to compete with the 24 h required to obtain a result with IHC [54].

The arrival of immune-oncology and of treatment targeting PD-L1/PD1 has substantially modified the care of patients with advanced stage or metastatic NSCLC. PD-L1 IHC is performed systematically by most laboratories for administration of first-line pembrolizumab therapy, even before obtaining the results of the genetic status of *EGFR*, *ALK*, *ROS1* and *BRAF*. Only patients without mutations in these four genes and showing expression of PD-L1 in more than 50% of tumor cells are eligible for treatment with pembrolizumab [63]. This raises the question of whether it is preferable to evaluate PD-L1 by IHC on the first tissue section and to then look for genomic alterations in the four genes or the reverse, knowing that the tissue surface for analysis is diminished and that the percentage of tumor cells for IHC analysis is reduced. Moreover, in some samples, less than 100 tumor cells are detectable (Figure 4E,F). Even if these latter tumor cells are strongly stained with PD-L1 antibodies, the significance of having only few positive tumor cells is questionable since the guidelines recommend to get more than 100 tumor cells for PD-L1 assessement.

The development of novel targeted therapeutic strategies for SCLC will potentially require evaluation by MB approaches. The degraded state of DNA often obtained for this histological type of lung cancer and the small-sized biopsies usually obtained by endoscopy, render difficult MB approaches. Thus, IHC is possibly more appropriate for detection of the expression of some molecules of interest.

## 5. Which Algorithms? For Which Patients? For Which Samples?

The increase in the number of therapeutic targets for detection on smaller and smaller samples has led to the development of algorithms for use with IHC in combination with MB techniques [8,45,107,108]. Irrespective of the algorithm they must integrate different parameters that can be modified from case to case. The size of the sample and the percentage of tumor cells must be obtained since this information can limit the use of certain MB techniques [18]. Thus, if the sample contains only a few tumor cells IHC is the preferred approach (for example for evaluation of the *ALK* and *BRAF V600E* status). Likewise, for PD-L1 IHC the first sections of small-sized biopsies should be used to examine the largest number of tumor cells possible. Ideally, a combined analysis looking for genomic alteration in *EGFR*, *ALK*, *ROS1* and *BRAF* is indispensable, and if considered necessary, also in *MET*, *RET*, *HER2*, and *NTRK*. If several biopsies are obtained inclusion into separate paraffin blocks of each biopsy may be recommended so as to conserve sufficient tissue material for both molecular analyses and IHC. Analysis by NGS can be doomed to failure under the circumstances described above. Before performing analyses, the sequential analysis by MB and IHC for detection of a target of interest must be discussed. It is then possible to look for mutations in *EGFR* on tissue sections, using molecular approaches that cover the majority of mutations, and to then evaluate the status of *ALK*, then *ROS1*, and finally BRAF V600E by IHC. In the case of a positive ROS1 IHC, confirmation by *ROS1* FISH should be performed.

These algorithms must take into account the information provided by the clinician (urgent need for the analyses, epidemiological parameters including the smoking history, age and the country of origin of the patient) [6,109]. In fact, it is possible that some of the collected data such as long-term exposure to tobacco and the age of the patient might limit certain analyses (in particular evaluation of a genomic modification in *EGFR*, *ALK* and *ROS1* which are rarer in heavy smokers), depending on the size of the tissue sample. Finally, these algorithms and the integration of both IHC and MB approaches should take into consideration the size of the sample, the percentage of tumor cells and the quality of the material provided to the laboratory. Even if some technology, such as the Sanger method can be still use by some center, it seems that there is no longer interest to develop this approach for tissue biopsies, due to its low sensitivity. Beside “personalized medicine”, we need to keep in mind that there is also probably “a personalized sample management”. In this context, regardless of the algorithms that are setup in a laboratory, the surgical and molecular pathologist should take into consideration each individual sample according to the different parameters described above and quite often there is a gap between the “real life in routine practice” and some well-established algorithms.

## 6. Perspectives and Conclusions

MB and IHC are complementary for care of patients with IIIB/IV stage lung cancer. The aim of these approaches is to define the molecular targets of interest and to thus rapidly propose effective first-line treatments as alternatives to conventional chemotherapy. The administration of immunotherapy or a therapy targeting molecular anomalies is the now the priority of the thoracic oncologist. However, the tissue specimens and/or cytological samples obtained do not always allow evaluation of all the molecular targets and different strategies are needed depending on the case, the clinical data and the epidemiology. In addition to the evaluation of the percentage of tumor cells labeled with PD-L1 IHC, the combined development of the analysis of the tumor mutational load and the microsatellite stability status can also modify rapidly the strategy [110,111,112]. It would then be a question of systematically looking for molecular biomarkers taking into account the size of the sample obtained by the pathology laboratory. The development of multiplex IHC approaches offers promising prospects to better economize the biological material of interest [113,114,115,116]. Thus, it would be ideal from the onset to combine evaluation of labeling with anti-PD-L1, ALK, ROS1 and BRAFV600E antibodies on a single tissue section and then detection by MB of different mutations in *EGFR* on a second tissue section, except in the case of patients included in therapeutic trials. Validation of the different clones on cytological samples should be performed before potentially using multiplex approaches for ICC [22]. Discussion into MB and IHC/ICC approaches using tissues or cells should examine rapidly the adoption of the technical advances made in the detection of therapeutic targets identified with liquid biopsies from the same patients [117,118,119]. While the tissue biopsy approach remains the gold standard it is most likely that complementary research will be performed with blood samples, either in a simultaneous or sequential fashion, depending of the results obtained with tissues [120,121,122] (Figure 5). Liquid biopsy can allow to look for different genomic alterations, but also may help for the detection of different biomarkers of interest expressed on circulating tumor cells by using ICC approaches [123] (Figure 5).
